# In Vitro Comparison of Lymphangiogenic Potential of Hypoxia Preconditioned Serum (HPS) and Platelet-Rich Plasma (PRP)

**DOI:** 10.3390/ijms24031961

**Published:** 2023-01-19

**Authors:** Jun Jiang, Xiaobin Cong, Sarah Alageel, Ulf Dornseifer, Arndt F. Schilling, Ektoras Hadjipanayi, Hans-Günther Machens, Philipp Moog

**Affiliations:** 1 Experimental Plastic Surgery, Clinic for Plastic, Reconstructive and Hand Surgery, Klinikum Rechts der Isar, Technische Universität München, D-81675 Munich, Germany; 2 Department of Plastic, Reconstructive and Aesthetic Surgery, Isar Klinikum, D-80331 Munich, Germany; 3 Department of Trauma Surgery, Orthopedics and Plastic Surgery, Universitätsmedizin Göttingen, D-37075 Göttingen, Germany

**Keywords:** peripheral blood cells, blood-derived therapy, hypoxia, lymphangiogenesis, wound healing, hypoxia preconditioned serum, platelet rich plasma, regenerative medicine

## Abstract

Strategies for therapeutic lymphangiogenesis are gradually directed toward the use of growth factor preparations. In particular, blood-derived growth factor products, including Hypoxia Preconditioned Serum (HPS) and Platelet-rich Plasma (PRP), are both clinically employed for accelerating tissue repair and have received considerable attention in the field of regenerative medicine research. In this study, a comparative analysis of HPS and PRP was conducted to explore their lymphangiogenic potential. We found higher pro-lymphangiogenic growth factor concentrations of VEGF-C, PDGF-BB, and bFGF in HPS in comparison to normal serum (NS) and PRP. The proliferation and migration of lymphatic endothelial cells (LECs) were promoted considerably with both HPS and PRP, but the strongest effect was achieved with HPS-40% dilution. Tube formation of LECs showed the highest number of tubes, branching points, greater tube length, and cell-covered area with HPS-10%. Finally, the effects were double-validated using an ex vivo lymphatic ring assay, in which the highest number of sprouts and the greatest sprout length were achieved with HPS-10%. Our findings demonstrate the superior lymphangiogenic potential of a new generation blood-derived secretome obtained by hypoxic preconditioning of peripheral blood cells—a method that offers a novel alternative to PRP.

## 1. Introduction

The peripheral lymphatic system consists of a complex network of lymphatic vessels that connect local tissue sites with secondary lymphatic organs [1]. In wound healing, lymph vessels play a critical role in interstitial fluid transport, which reduces excessive edema and supports angiogenic, inflammatory, and proliferative processes, thus contributing to more efficient tissue regeneration [2]. Dysfunction of the lymphatic flow can lead to lymphedema, which is characterized by the disruption of the immune clearance of affected sites, an increase in bacterial colonization, and the entrapment of growth factors and other matrix proteins [1,3]. These factors contribute to halting wound healing processes at the inflammation stage, which can cause a further decline in lymphatic function and eventually delay tissue repair [4]. Several experimental studies suggest that failed or insufficient lymphangiogenesis can be one possible cause of impaired wound healing [3,5]. Thus, restoration of the lymphatic system is reported as imperative for an appropriate healing response and has consequently developed into a major target for the treatment of chronic non-healing wounds [2,6].

In contrast to blood vessel angiogenesis, lymphangiogenesis has not been emphasized enough as a key player in the process of wound healing. Nonetheless, there is a need for further research on the biomolecular mechanisms of lymphatic regeneration after skin injury and of failed lymph vessel generation in non-healing wounds [7]. To date, we have elucidated that the functional processes of angiogenesis and lymphangiogenesis are intertwined but are also markedly different, commencing at different times and responding to different biochemical stimuli [2,7]. In wound healing, repair of the lymphatic network follows at a later stage than angiogenesis [8] and is induced by alternate factors, including vascular endothelial growth factors (VEGF)-C and D, which activate vascular endothelial growth factor receptor 3 (VEGFR-3), the primary mediator of lymphangiogenesis [2,9,10]. Angiogenesis-related growth factors, such as basic fibroblast growth factor (bFGF), insulin-like growth factor 1/2 (IGF-1, IGF-2), hepatocyte growth factor (HGF), endothelin-1 (ET-1), and platelet-derived growth factor-BB (PDGF-BB) have also been reported to induce lymphangiogenesis, whereas thrombospondin-1 (TSP-1), platelet factor-4 (PF-4), and endostatin have been demonstrated as key endogenous inhibitors of lymphangiogenesis [7]. These biomolecules are produced by activated macrophages, T cells, mast cells, and dendritic cells and can be released by proteolysis of molecules of the extracellular matrix in the wound bed [7,11].

Several studies have explored therapeutic lymphangiogenesis as a potential means to improve the healing of diabetic wounds, which are challenging to treat and tend to develop high amounts of interstitial fluids [12,13]. In models where lymphatic vessels were ablated, local delivery of recombinant VEGF-C was shown to improve healing and reduce tissue edema [12,14]. These results give credence to the therapeutic potential of VEGF-C. Unfortunately, VEGF-C can also induce the pathological permeability and hyperplasia of blood vessels as well as dysfunctional remodeling of collecting lymphatic vessels. Therefore, repeated administration of high-dose VEGF-C as a single growth factor therapy can lead to edema and venous enlargement [7,15,16]. Conclusively, a more encompassing growth factor preparation, also containing regulatory lymphangiogenic growth factors, would be a better tool for the natural restoration of tissue integrity.

In this context, blood-derived growth factor preparations present a biologically viable and easily obtainable source of an all-inclusive preparation, which has received much attention in the field of regenerative medicine [17,18,19]. We developed a novel approach of using hypoxia, one of the primary stimuli of angiogenesis/lymphangiogenesis in wound healing, to stimulate peripheral blood cells (PBCs) to produce angiogenic and lymphangiogenic cytokines [20,21,22,23]. PBCs are ideal providers of physiological regenerative signals, which can be obtained on-demand using specific stress-induced treatment (e.g., hypoxia) [17,20,21,22,23,24,25,26,27,28,29,30,31,32]. Our method utilizes extracorporeal hypoxia-adjusted preconditioning by cultivating PBCs within a self-regulated low-oxygen microenvironment. During hypoxic incubation, PBCs sediment and release biomolecules into the serum compartment (Hypoxia Preconditioned Serum: HPS), which can be separated from the blood cells through filtration without the need for centrifugation, as required in the Platelet-rich Plasma (PRP) preparation [17,20,22,24,27]. We have previously proved that angiogenic growth factors, such as VEGF (especially VEGF-C), bFGF, IL-8, and MMP-9 [17,20,24,33], as well as anti-angiogenic factors such as TSP-1 and PF-4 [17,20,24,31], are strongly upregulated in HPS in comparison to unstimulated serum. Upon the examination of hypoxia-induced secretomes for their angiogenic and lymphangiogenic activity, we were able to illustrate their ability to promote microvessel formation and sprouting in vitro, as well as accelerate natural wound healing in vivo [17,20,24,29].

PRP is currently perceived as the gold standard of blood-based regenerative therapy [19]. It has been safely used and documented in different fields, including orthopedics, dental, plastic, cardiovascular, and maxillofacial surgery, and has drawn attention to promoting tissue remodeling and wound healing [19,34,35,36,37]. In comparison to HPS, growth factor release in PRP relies only on the activation of platelets, which are concentrated through centrifugation to a supraphysiological level (up to six times more than in peripheral blood) [18,24]. Degranulation and release of their pre-stored biomolecules form a different balance of pro- and anti-angiogenic/lymphangiogenic growth factors than in HPS [24,26], which constitutes the platelet-derived secretome, including similar key growth factors, such as VEGF, PDGF, bFGF, IGF-1, TSP-1 and PF-4 [24,38,39]. In this regard, HPS comprises not only the platelet-derived factors that are released during the blood clotting phase but also the complete secretome of growth factor proteins that are produced by PBCs during the inflammatory and angiogenic/proliferative phases of wound healing [24]. The relevance of HPS in lymphatic regeneration has been demonstrated previously, both in vitro and in vivo, but there is insufficient research on the lymphangiogenic potential of PRP and no comparison data of the two secretomes [26,29,40].

In this study, we aim to comprehensively investigate the contribution of the hypoxia-induced secretome of HPS to lymphangiogenesis in comparison to the platelet-derived secretome of PRP and normal (non-hypoxia-induced) serum (NS). Initially, a proteomic lymphangiogenic growth factor analysis of the secretomes was performed, followed by the investigation of the lymphatic regeneration potential using human lymphatic endothelial cells (LECs). In the next stage, we used HPS and PRP in a more complex 3D model of an ex-vivo lymphatic ring sprouting assay. With this study, we aim to develop a new generation tool with HPS that bio-actively boosts natural tissue regeneration.

## 2. Results

### 2.1. Quantitative Analysis of Pro- and Anti-Lymphangiogenic Growth Factors in Different Human Blood-Derived Secretomes

**Figure 1:**
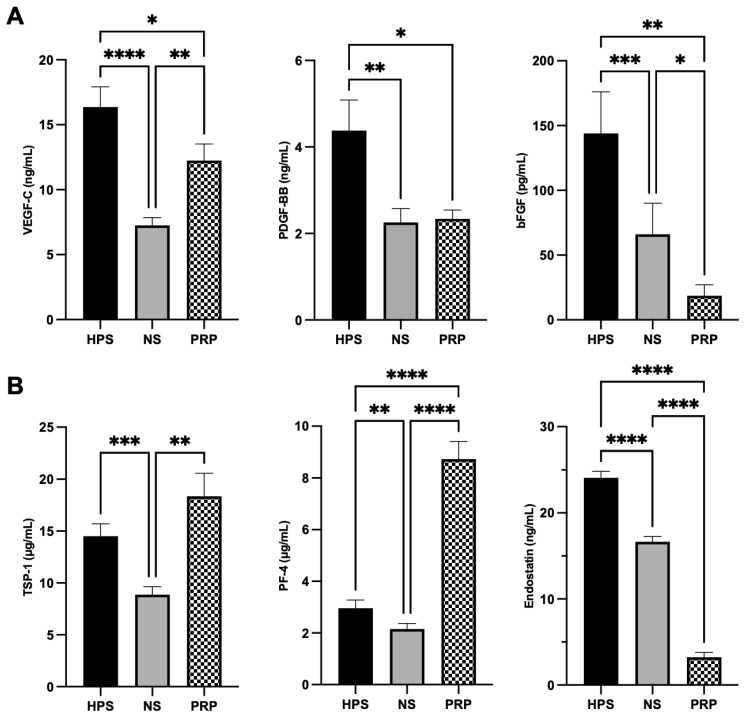
Quantitative analysis of pro- and anti-lymphangiogenic growth factors in HPS, NS, and PRP. (**A**) Protein quantification of the key pro-lymphangiogenic growth factors VEGF-C, PDGF-BB, and bFGF. HPS showed significantly higher VEGF-C, PDGF-BB, and bFGF levels than NS and PRP (*p* < 0.05). (**B**) Protein quantification of the key anti-lymphangiogenic growth factors TSP-1, PF-4, and endostatin. One-way repeated-measures ANOVA with Tukey’s multiple comparison test. Data points are means ± SEM, blood donors: *n* = 9. * *p* < 0.05, ** *p* < 0.01, *** *p* < 0.001, **** *p* < 0.0001.

In the first step of characterizing HPS, NS, and PRP, we quantitatively analyzed the concentration of three pro- (VEGF-C, PDGF-BB, and bFGF) and three anti-lymphangiogenic (TSP-1, PF-4, and endostatin) growth factors in the blood-derived secretomes. For PRP-secretome evaluation, we used the releasate of activated concentrated platelets, which reached a mean of 4.91× higher platelet concentration than in whole blood (1213.33 ± 126.66 vs. 246.66 ± 38.44 × 10^9^/L). All three pro-lymphangiogenic growth factors (VEGF-C, PDGF-BB, and bFGF) were significantly increased in HPS compared to both NS and PRP (*p* < 0.05) (Figure 1A). PRP contained more VEGF-C (*p* < 0.01) but less bFGF (*p* < 0.05) compared to NS. When examining the anti-lymphangiogenic factors, TSP-1 was comparably elevated in both HPS and PRP in comparison to NS (*p* < 0.001 and *p* < 0.01, respectively) (Figure 1B). PF-4 was strongly increased in PRP compared to HPS and NS (both *p* < 0.0001), and HPS showed mildly elevated PF-4 levels in comparison to NS (*p* < 0.01). The highest concentration of endostatin was measured in HPS (*p* < 0.0001 vs. NS and PRP). All these results were gender-independent.

### 2.2. The Effect of Different Human Blood-Derived Secretomes on the Proliferation and Migration of LECs

**Figure 2:**
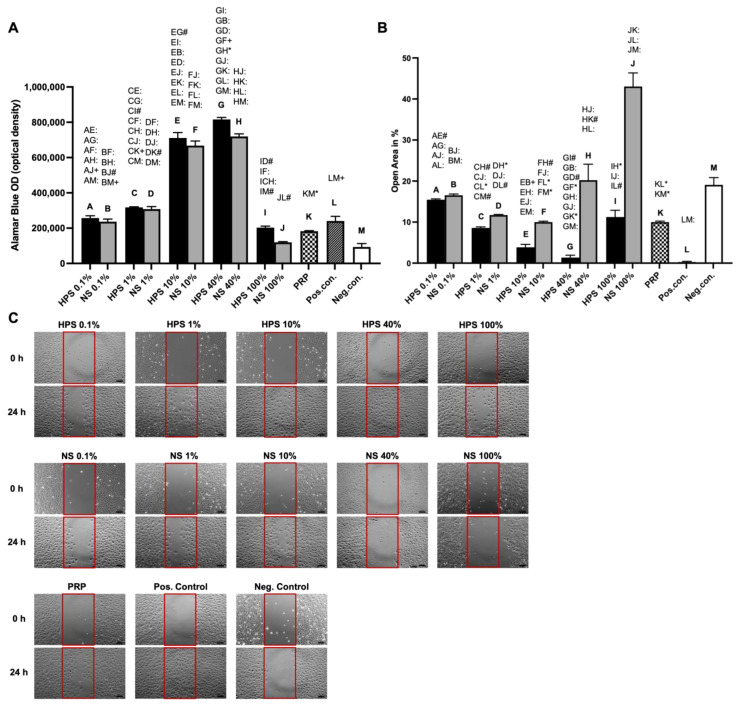
The effect of HPS, NS, and PRP on proliferation and migration of LECs. LECs were stimulated by HPS/NS-0.1%, -1%, -10%, -40%, 100%, and PRP (pooled from ten donors) compared to positive/negative control. (**A**) Plot showing Alamar Blue proliferation assay measured in optical density (OD) after 96 h of stimulation. (**B**) Plot showing closure of the open area (residual area in % of the full area) after 24 h calculated by image analysis of the digital photographs depicted in (**C**). (**A**,**B**): One-way repeated-measures ANOVA with Tukey’s multiple comparison test. Data points are means ± SEM, LEC donors: *n* = 3. Capital letter pairs over plots indicate statistical comparison of corresponding data points. For all pair comparisons, * = *p* < 0.05, # = *p* < 0.01, + = *p* < 0.001, : = *p* < 0.0001. (**C**) Representative microscopic photographs of scratch assay of LECs (*n* = 3) stimulated by the blood-derived secretomes, taken at 0 h and 24 h. Red boxes indicate the initially standardized open area. Scale bar = 100 μm.

In the next series of experimental testing, we evaluated the proliferative and migrative effects of blood-derived secretomes on LECs. We analyzed different HPS dilutions to determine the most beneficial HPS concentration, thus alternating the net effect of the pro- and anti-lymphangiogenic growth factors and comparing them to corresponding dilutions of NS. The PRP-secretome remained undiluted, as this is, per definition, a concentrated cocktail of platelet-derived growth factors. For proliferation assessment, we used the Alamar Blue assay, which detects viable cells through a mitochondrial reduction reaction. Here, the highest proliferation was achieved by increasing the concentration of HPS to 40%, which was considerably higher than all other concentrations of HPS and NS (especially NS-40%) (*p* < 0.01), and outperformed PRP by 4.5× and even the positive control (expansion medium) by 3.4× (both *p* < 0.0001) (Figure 2A). Lower concentrations of HPS (HPS-0.1/1/10%) showed higher mean values than their corresponding NS (NS-0.1/1/10%), but these comparisons were not statistically significant (*p* > 0.05). Cell proliferation with HPS-100% was decreased and comparable to PRP, which was, in turn, comparable to the positive control and, interestingly, inferior to NS-1/10/40% (*p* = 0.0012, *p* < 0.0001, *p* < 0.0001, respectively).

The migration of LECs is critical for vessel sprouting and remodeling in lymphangiogenesis [41] and was therefore assessed using a scratch assay by monitoring a denuded open area. We were able to demonstrate that migration was promoted similarly to the proliferation assay dose-dependently with a higher HPS concentration of up to 40% (smaller open area) but decreased at a concentration of 100% (Figure 2B,C). HPS-40% had a higher migration capacity in comparison to both PRP (open area: 1.32 ± 0.60% vs. 10.00 ± 0.23%, *p* = 0.03) and NS-40% (open area: 1.32 ± 0.60% vs. 20.23 ± 3.90%, *p* < 0.0001). Lower concentrations of HPS (HPS-0.1/1/10%) showed smaller mean open areas than their corresponding NS (NS-0.1/1/10%), but these comparisons were not statistically significant. Interestingly, diluted NS (NS-0.1%, 1%, and 10%) had a similar effect on migration as with PRP (Figure 2B,C).

### 2.3. The Effect of Different Human Blood-Derived Secretomes on Tube Formation of LECs

**Figure 3:**
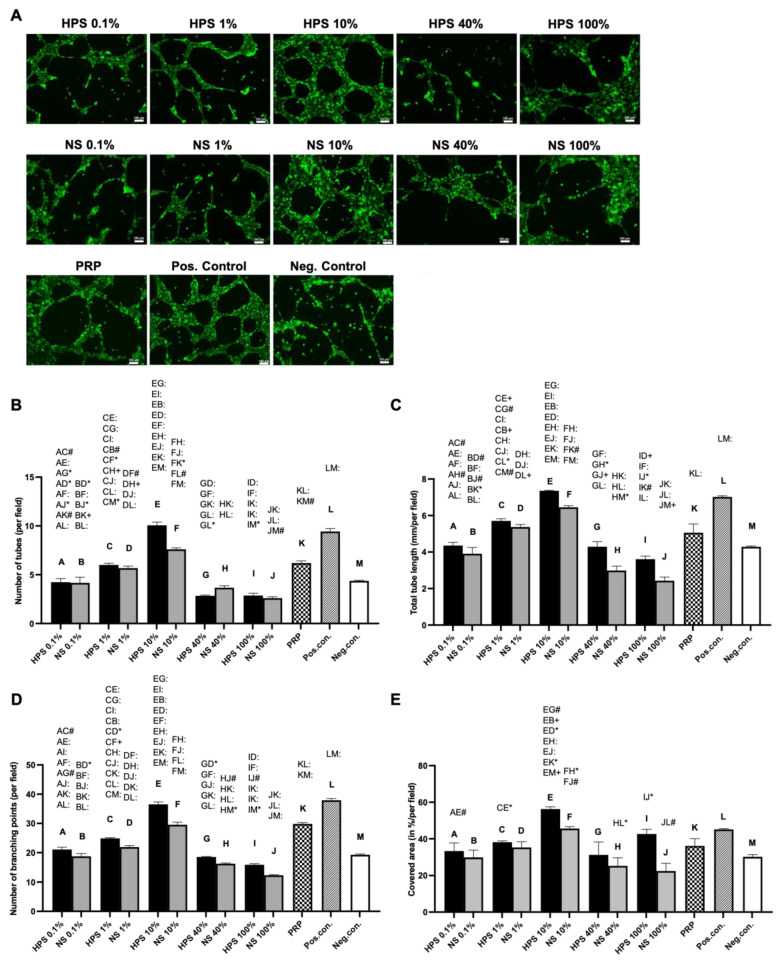
The effect of HPS, NS, and PRP on tube formation in LECs. (**A**) Representative microscopic photographs of the tube formation assay (8 h) of LECs stained by Calcein AM, carried out in the presence of the shown blood-derived secretomes (pooled from ten donors) compared to positive/negative control. Scale bar = 100 μm. (**B**–**E**) Image analysis of the digital photographs depicted in (**A**): (**B**) Plot showing the number of tubes. (**C**) Plot showing total tube length. The length is measured in mm. (**D**) Plot showing the number of branching points. (**E**) Plot showing percentage of the covered area of LECs. One-way repeated-measures ANOVA with Tukey’s multiple comparison test. Data points are means ± SEM, LEC donors: *n* = 3. Capital letter pairs over plots indicate statistical comparison of corresponding data points. For all pair comparisons, * = *p* < 0.05, # = *p* < 0.01, + = *p* < 0.001, : = *p* < 0.0001.

In addition to the proliferation and migration of LECs, the ability of LECs to form vessel-like structures under stimulations of HPS, NS, and PRP was assessed via tube formation assay. In short, LECs were seeded at a sub-confluent density with extracellular matrix support to form capillary-like structures in order to evaluate the tube-forming capabilities of the secretomes. Data analysis parameters included the number and the total length of tubes, the number of branching points, and the percentage of cell-covered area. LECs treated with HPS-10% developed the highest number of tubes, greater than both the PRP (10.07 ± 0.32 vs. 6.20 ± 0.21, *p* < 0.0001) and NS-10% groups (10.07 ± 0.32 vs. 7.60 ± 0.15, *p* < 0.0001) (Figure 3B). Furthermore, HPS-10% also displayed a higher number of tubes in comparison to both lower concentrations of HPS (HPS-0.1%, HPS-1%) and higher concentrations of HPS (HPS-40%, HPS-100%) (*p* < 0.0001). The total tube length was also higher with HPS-10%-stimulation in comparison to PRP (7.35 ± 0.03 mm vs. 5.06 ± 0.48 mm, *p* < 0.0001) (Figure 3C). In contrast to NS-10%, tube lengths of HPS-10% treated cells were higher albeit not significantly (7.35 ± 0.03 mm vs. 6.45 ± 0.09, *p* > 0.05). The results of the total branching points concurred with the total tube number, with HPS-10% promoting more points than PRP (36.53 ± 0.79 vs. 29.83 ± 0.44, *p* < 0.0001) and NS-10% (36.53 ± 0.79 vs. 29.53 ± 0.91, *p* < 0.0001) (Figure 3D). With regard to the cell-covered area, the HPS-10% treated LECs displayed the greatest cell-covered percentage, which was also significantly greater than PRP (56.27 ± 1.27% vs. 36.09 ± 3.94%, *p* = 0.0177) (Figure 3E). Higher concentrations of HPS (HPS-40% and 100%) showed a lower cell-covered percentage (31.19 ± 7.04% and 42.63 ± 2.57%, respectively). In summary, the increasing and decreasing trends in HPS-treated groups were similar regarding the total tube number, length, branching points, and cell-covered area, which peaked at HPS-10% and decreased towards either HPS-0.1% or HPS-100%. Therefore, a concentration of HPS-10% was found to be supportive of LEC tube formation and of higher efficiency than the PRP treatment.

### 2.4. The Effect of Different Human Blood-Derived Secretomes on Ex Vivo Lymphatic Ring Sprouting

**Figure 4:**
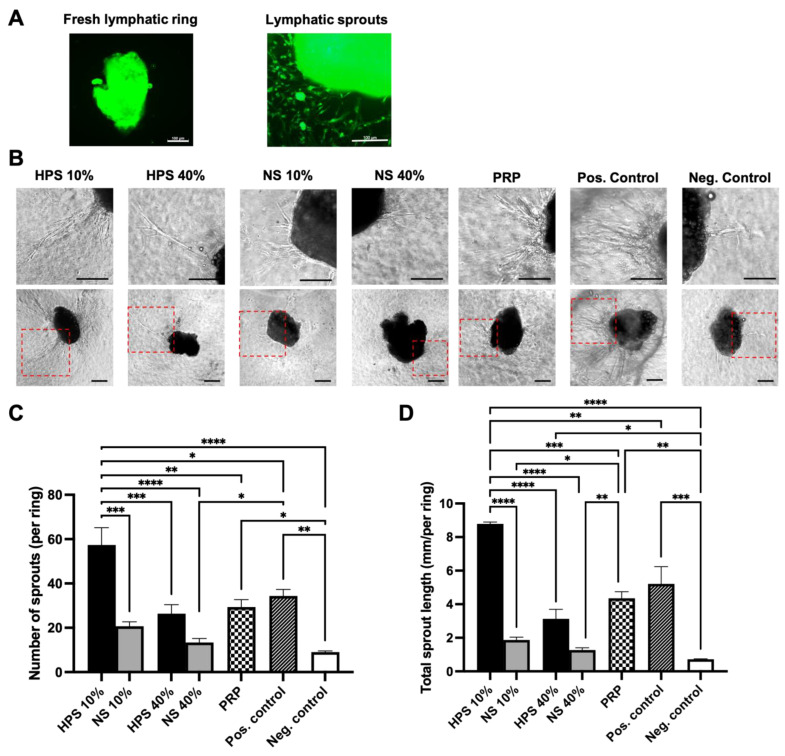
The effect of HPS, NS, and PRP on ex vivo lymphatic ring sprouting. (**A**) Validation of the lymphatic ring assay: Representative image of immunohistochemical staining of a thoracic duct ring with Anti-LYVE-1 (green fluorescence) before culture (left) and validation of lymphatic origin of the sprouts after 11 days with HPS-10%-stimulation (right). Scale bars = 100 µm. (**B**) Panel showing representative images of lymphatic ring cultures (11 days culture), stimulated by HPS/NS-10%, -40%, and PRP compared to positive/negative control (second row). The first row demonstrates enlarged images of sprouts from the dotted red boxes of the second-row panel. Scale bars = 100 µm. (**C**,**D**) Image analysis of the digital photographs depicted in (**B**): (**C**) Plot showing the number of sprouts and (**D**) total sprout length of thoracic ducts. The length was measured in mm of the digitally captured microscopic photographs. One-way repeated-measures ANOVA with Tukey’s multiple comparison test. Data points are means ± SEM, thoracic duct donors: *n* = 3. * *p* < 0.05, ** *p* < 0.01, *** *p* < 0.001, **** *p* < 0.0001.

The three-dimensional lymphatic ring assay phenotypically emulates the essential steps of lymphangiogenesis, including sprouting from a pre-existing vessel, cell proliferation, migration, and forming into capillaries [42]. It also bridges the gap between two-dimensional in vitro models, such as the previously shown LEC tube formation assay, and in vivo models of lymphangiogenesis [42]. Firstly, we verified the lymphatic origin of the harvested tissue and vessel sprouts by immunohistochemical anti-lymphatic-vessel-endothelial-hyaluronan-receptor-1 (anti-LYVE-1)-staining (Figure 4A). In our previous LEC migration and tube formation assays, we identified beforehand HPS-10% and HPS-40% as optimal secretome concentrations for lymphangiogenesis (see Section 2.2 and Section 2.3). Hence, both concentrations were used for the ex vivo lymphatic ring assay, in addition to NS-10%, -40%, and PRP. Then, the total number and length of sprouts were analyzed via digital photography. Here, we were able to confirm the superior lymphangiogenic capability of HPS-10% in comparison to all the other conditions, explicitly NS-10%, HPS-40%, PRP, and the positive control (Figure 4B,C). HPS-10% yielded more than double (2.8×) the sprouting number than NS-10% (57.33 ± 7.84 vs. 20.67 ± 2.03, *p* = 0.0002) and 1.95× more than PRP (57.33 ± 7.84 vs. 29.33 ± 3.38, *p* = 0.0025) and even was significantly greater than the positive control (57.33 ± 7.84 vs. 34.33 ± 2.96, *p* = 0.0127). HPS-10% also gained the greatest sprout length correspondingly: 4.7× compared to NS-10% (8.79 ± 0.10 mm vs. 1.87 ± 0.17 mm, *p* < 0.0001), 2.0× compared to PRP (8.79 ± 0.10 mm vs. 4.35 ± 0.40 mm, *p* = 0.0002) and 1.7× compared to the positive control (8.79 ± 0.10 mm vs. 5.21 ± 1.03 mm, *p* = 0.0017). PRP’s sprout generation (number and length) was inferior to HPS-10%; however, it was comparable to HPS-40% and the positive control.

## 3. Discussion

Wound repair is a complex system of biological interactions and involves the coordination of numerous cell types and biomolecules and the restoration of the vascular system [43]. Current strategies of accelerating tissue regeneration are directed towards biological tissue replacement, (stem-) cell-based treatments, gene therapy, and the exogenous supply of recombinant angiogenesis factors [44]. In this context, research on regenerative therapies has been predominantly focused on the promotion of blood vessel vascularity, which certainly represents a key driver of tissue repair [2]. Furthermore, thinking beyond this concept, complete restoration of tissue integrity requires the recovery of interstitial fluid drainage and thus necessitates the regeneration of lymphatic vessels, which needs to be considered as a fundamental process in wound healing, complementary to angiogenesis [7].

In regenerative therapies using blood-derived growth factors, PRP is considered the gold standard and has been increasingly utilized over the past decades since it delivers a natural source of various on-demand growth factors that are pre-stored in the platelet granules [35,37,39,40]. In the further development of autologous growth factor preparations, we established a newer method using hypoxic preconditioning of PBCs, which exploits the wound healing phases from hemostasis, and inflammation to the angiogenesis-driven proliferative phase, which differs from the PRP preparation involving only the hemostasis phase [17,20,21,24,31]. In this aspect, we hypothesized a benefit of HPS over PRP, especially in lymphangiogenesis, since physiologically, these processes begin at a later stage of wound healing, following angiogenesis [8]. The promotion of lymphatic regeneration by HPS has been previously demonstrated in a lymphatic ring assay and, more recently, in an in vivo murine wound healing experiment in which lymphatic vessels are strongly upregulated in the wound bed with HPS treatment [26,29]. However, the lymphangiogenic effect of the platelet-derived secretome has not been conclusively evaluated, and no investigation has been conducted regarding the effect on LECs. In this study, our investigations demonstrate a substantial lymphangiogenic response to both HPS and PRP treatment, most probably by providing lymphangiogenic growth factors, the consequent promotion of proliferation and migration of LECs and microvessel formation of both LECs and lymphatic duct vessels. HPS outperformed PRP in its lymphatic regenerative capacity in all those experiments. This may allow designing a new generation of growth factor treatments that pushes current regenerative medicine research forward in realizing a complete and natural restoration of tissue integrity.

We have come to understand that the biomolecular mechanisms responsible for lymphangiogenesis are distinct from those of angiogenesis and depend primarily on the binding of VEGF-C/D rather than VEGF-A/B [45]. In this study, we elected biomolecules for the proteomic analysis, which are both regulated via hypoxia/stress-induced PBCs and platelet-activation (VEGF-C, PDGF-BB, bFGF, TSP-1, PF-4, and endostatin) [17,20,24,26,46,47]. We were able to discern fundamental differences in the key pro- (VEGF-C, PDGF-BB, bFGF) and anti-lymphangiogenic (TSP-1, PF-4, endostatin) growth factors in both HPS- and PRP-secretomes (Figure 1). Hypoxia-preconditioning of PBCs elicited higher levels of pro-lymphangiogenic VEGF-C, PDGF-BB, and bFGF in comparison to PRP and the baseline NS. VEGF-C, the key player of lymphangiogenesis, was upregulated in HPS up to 2.3× higher than in NS and up to 1.4× higher than in PRP (Figure 1A). Interestingly, the PDGF-BB level in PRP (containing 4.9× more platelets than in whole blood in our experiments) was lower than HPS and comparable to NS, which comprises a secretome also mainly based on activated platelets through blood clotting. Studies revealed that fresh serum, which was slowly prepared (sedimentation for 24 h at 4 °C), promoted considerable growth factor release in platelets, such as PDGF, compared to rapid preparations (30 min at 20 °C) [48]. Since our protocol required sedimentation for 4 h at 20 °C, the elevated release of factors (bFGF and endostatin) in NS can thus be explained. Upon the examination of anti-lymphangiogenic growth factors, HPS and PRP were found to contain disproportional amounts of the investigated biomolecules, which makes the total lymphangiogenic effect difficult to predict: TSP-1 was comparable between HPS and PRP, but PF-4 was (as expected) 3.0× higher in PRP than in HPS, and endostatin was 7.5× higher in HPS than in PRP (Figure 1B). In this regard, higher levels of (lymph-)angiogenesis inhibitors (TSP-1, PF-4) in HPS have previously been demonstrated not to cause inferior lymph vessel generation in comparison to Hypoxic Preconditioned Plasma (HPP), a product that lacks platelet activation through the addition of heparin anti-coagulation, resulting in lower TSP-1 and PF-4 concentration than in HPS [24]. Accordingly, the regulation of lymphangiogenesis requires more than the investigation of a measurable balance between pre-selected pro- and anti-lymphangiogenic factors of secretomes. Therefore, additional assays (LEC proliferation, migration, tube formation, and lymphatic ring assay) were performed in this study to estimate the lymphangiogenic differences between HPS and PRP. However, their ultimate promoting effects on LECs need to be analyzed in future experiments regarding the expression of lymphatic markers of LECs, e.g., VEGFR-3, endogenous VEGF-C, or transcription factor Prox-1, to achieve deeper insights into the mechanisms of action [7,49].

Lymphangiogenesis is described to occur in the process of endothelial cell proliferation, migration, and forming of three-dimensional vessels [7]. Firstly, we investigated the proliferative effects of the blood-derived secretomes on LECs. With the objective of elucidating a clinically beneficial concentration of HPS and adjusting the effect of the pro- and anti-lymphangiogenic factors, we diluted the HPS secretome serially from 100% to 40%, 10%, 1%, and 0.1%. For PRP-secretome evaluation, we used the releasate of activated concentrated platelets dissolved in the culture medium. Dilution of PRP was deliberately avoided, as it is, per definition, a concentrated secretome (i.e., dilution of activated PRP would resemble the secretome of normal serum). Here, we demonstrated better proliferative capacity with increasing concentrations of HPS up to 40% (Figure 2A). Particularly HPS-40% showed the highest proliferation when compared to all the other dilutions of HPS and NS, and especially compared to PRP (up to 4.5×) and the positive control (up to 3.4×). Nevertheless, HPS-10% already demonstrated a substantial promotion of proliferation, which was 13% less than HPS-40% (Figure 2A). With regard to migration analysis, the results indicate similarly that higher concentrations of HPS up to 40% promote LEC migration dose-dependently. Particularly HPS-40% demonstrated again noticeably higher migration compared to NS-40% (up to 15.3×) and PRP (up to 7.6×) (Figure 2B). These results reveal the positive effect of hypoxic preconditioning of PBCs and the lymphangiogenic potential of HPS, which is also in accordance with the previously demonstrated positive effects of HPS in blood vessel angiogenesis [17,24,26]. With higher HPS concentration towards 100%, the culture medium is deprived and progressively replaced by serum. This may be a reason why LECs declined in their proliferative and migrative function. With PRP, we demonstrated, for the first time, that LEC proliferation and migration were considerably elevated in comparison to the negative control but were inferior to HPS. A direct comparison of PRP to pure HPS (=HPS-100%) is not fully eligible, although PRP outperformed HPS-100% in migration since PRP contained released growth factors dissolved in culture media and HPS is technically a product of growth factors dissolved in serum. We may expect even more optimized LEC proliferation and migration if the HPS secretome could be released into the fresh culture medium; however, this is not possible due to the four-day process of hypoxic preconditioning.

Subsequently, we investigated the effect of HPS, PRP, and NS on microvessel generation in LEC cultures using the tube formation assay. Here, we discovered increased lymphatic tube formation (number of tubes, tube length, number of branching points, covered area) with lower HPS concentrations up to 10% and a decreased positive effect with concentrations over 10% (Figure 3). Interestingly, tube formation subsided in HPS-40%, which was presented previously as the ideal concentration for cell proliferation and migration. HPS-10% had a considerably stronger effect in tube formation than PRP and NS-10%, indicating once again the positive effect of hypoxic preconditioning of PBCs. Compared with the previous blood vessel angiogenesis results from our group, where the strongest angiogenic effect was demonstrated at HPS-1%, the LECs also elicited a similar bell-shaped lymphangiogenic response profile dependent on different HPS dilutions [17]. Choosing the ideal concentrations of HPS so far with 10% (tube formation) and 40% (cell proliferation/migration), we continued our investigations in a more complex lymphatic sprouting assay that summarizes the essential steps of lymphangiogenesis [7]. Tube formation assay of LECs reflects the generation of capillary-like structures from seeded cells, similar in embryogenesis, but mechanisms of in vivo lymphangiogenesis rely as well on the sprouting from pre-existing vessels. The question of which theory predominates in lymphangiogenesis has not been resolved to this day [2,7]. Consequently, we conducted an ex vivo organotypic culture of murine thoracicus duct vessels and examined the number and length of the sprouts after stimulation by the blood-derived secretomes. Results from the tube formation analysis were double-validated with the sprouting assay, which put HPS-10% noticeably at the forefront of all the other groups (NS-10%, HPS/NS-40%, PRP, and positive control). Remarkably, HPS-10% induced double the sprout number and length in comparison to PRP and was considerably more effective than the positive control (Figure 4B–D). Comparing these results to blood vessel angiogenesis in a murine aortic ring assay performed previously by our group, HPS-10% was evaluated as well as the optimal concentration [24]. Therefore, HPS-10% seems to be the overall strongest natural stimulant for the restoration of vascularity, i.e., angiogenesis and lymphangiogenesis. From a morphological point of view, there was no subjective difference in the sprout width from the root to the tip between the HPS, NS, and PRP-treated thoracic ducts (Figure 4B). Even though collagen type I turbidity was a limitation for visualizing sprouts in phase-contrast pictures, we were still able to analyze the sprouts of different depths by examining images at different microscopic focus levels. Meanwhile, immunostaining allowed us to determine the lymphatic origin of the tissue. Nevertheless, more comprehensive immunostaining of the sprouts has to be performed to analyze further differences in microvessel development, perivascular recruitment, and remodeling.

The surprising difference demonstrated here in the optimal dilution of the HPS-secretome between proliferation/migration and tube/sprout formation (HPS-40% vs. HPS-10%) promotes the understanding that to achieve the greatest micro-vessel formation, single steps of proliferation and migration might not present a full account in this process, and reaching the performance of HPS-10% is already ideal. From another perspective, the growth factor demand in the wound healing process is assumed to follow dynamic spatio-temporal actions, meaning different concentrations of biomolecules interact at different times (wound healing stages) and in different spaces (wound depths) during tissue regeneration [2,7,24,27]. Therefore, a higher growth factor demand in the initiation of lymphangiogenesis, which includes the steps of proliferation and migration of LECs, could be possible, followed by a lower growth factor demand in the process of lymphatic vessel generation, which is in accordance with other researchers’ findings on cytokine change in wound healing [50,51]. Indeed, all these processes might be differently regulated through the alternate balances of pro- and anti-angiogenic factors in the wound bed. Deciphering these complex interactions between the multitudes of growth factors, e.g., through the inhibition of key regulatory factors, is an essential endeavor that is yet to be undertaken. In this context, we have previously detected PF-4 as an important factor for endothelial detachment from pre-existing vessels, a process that is indispensable for new vessel generation in lymphangiogenesis [27,52]. Low levels of PF-4 are necessary for vessel sprouting, while higher levels of PF-4 inhibit angiogenesis [27,52]. Taking this into consideration, the high concentration of PF-4 in PRP (3.0× more than HPS, see Figure 1) may result in an inhibitory effect on the generation of new and specifically longer vessels, regardless of its pro-angiogenic VEGF levels. This theory has previously been verified by our group in relation to angiogenesis [24] and, subsequently, in this study, with the process of lymphangiogenesis. Both results do not seem surprising, as platelets play a key role in maintaining hemostasis during the early stages of wound healing and regulating/inhibiting the formation of immature or hemorrhagic vessels and the extension of vessel length [53,54]. Therefore, pure platelet-derived factors might not represent an ideal and/or comprehensive approach to regenerating tissue integrity in the long run. Accordingly, the basic notion of overconcentrating platelet-derived factors in PRP to effectively promote a (lymph-)angiogenic response in tissue regeneration is shadowed with doubt. More importantly, PRP failed to stimulate higher angiogenic and lymphangiogenic responses than diluted normal serum [24]. In contrast, utilization of the hypoxia-driven phases of wound regeneration following the hemostatic phase as a foundation to mimic all the steps of tissue regeneration might bring us closer to engineering physiologically supported wound healing. However, translating in vitro experiments into in vivo applications has to incorporate additional external factors; thus, further in vivo examinations of HPS and PRP effects should be conducted, especially regarding the effectiveness of the newly generated lymph vessels, e.g., in a murine tail lymphedema model [55].

## 4. Materials and Methods

### 4.1. Ethical Approval

This study was conducted per the Declaration of Helsinki and the approval of the ethics committee of the Technical University Munich, Germany (File Nr.: 497/16S; Amendment, date of approval: 11 November 2016). Informed consent was obtained from all blood donors involved.

### 4.2. Production of Hypoxia Preconditioned Serum (HPS)

Hypoxia Preconditioned Serum (HPS) was produced following the method previously described by our group [17]. The donor-selection criteria included: 10 healthy human donors (3 females/7 males) with an age distribution ranging from 20 to 34 years. The exclusion criteria included: smokers, pregnant donors, donors with systemic inflammatory diseases, and donors treated with oral medication within the last 6 weeks prior to donation. In short, 20 mL of peripheral venous blood was collected into a 30 mL syringe (Omnifix^®^, B Braun AG, Melsungen, Germany), and then 5 mL of air was drawn through a 0.2 µm filter (Sterifix^®^, B Braun AG, Melsungen, Germany). The syringe was subsequently sealed, creating a pericellular hypoxia (~1% O_2_) by PBCs’ oxygen consumption during an incubation period of 4 days at 37 °C and 5% CO_2_. Post-incubation, three distinct layers were formed, with the top ‘clear’ layer representing the HPS, which was filtered (Sterifix^®^, B Braun AG, Melsungen, Germany) into a new syringe for further pooled or individual aliquots at −80 °C until experimental testing (for a maximum of 3 months). Although the levels of VEGF decreased within the 3-month period, frozen-stored HPS secretomes demonstrated no negative impact on angiogenesis [17,24].

### 4.3. Production of Platelet-Rich Plasma (PRP)

The production of Platelet-rich Plasma (PRP) followed an established double-centrifugation protocol [56]. The donors were identical to those from the HPS group (see Section 4.2). Briefly, 6 mL of peripheral venous blood was collected into 6 mL-blood collection tubes (366575, BD Vacutainer, Becton, Dickinson and Company, Franklin Lakes, NJ, USA) prefilled with trisodium citrate and centrifuged at 1300× *g* for 20 min. The blood was then separated into platelet-poor plasma (top layer), buffy coat (middle layer containing platelets and white blood cells), and erythrocytes (bottom layer). The upper two layers, which account for 60% of the whole blood volume, were pipetted into a new falcon. To minimize any loss of platelets, a few erythrocytes beneath the buffy coat layer were permitted to be collected. A secondary centrifugation of 1800× *g* ensued for 15 min to separate the bottom PRP (approx. 0.5 mL) from the upper serum component. The serum component was then removed, and the PRP was activated by adding 0.5 mL of 1 I.U./mL Thrombin and 8.88 μg/mL CaCl_2_ (Tisseel, Baxter, Illinois, USA), which were solved in basal media (see Section 4.5). After incubating the mixture for 30 min at 37 °C, a third centrifugation was carried out at 2500× *g* for 20 min to attain an activated PRP supernatant, which is an equivalent of a releasate of the PRP-secretome dissolved in basal media. PRP was then collected by a sterile syringe and filtered (Sterifix^®^, B Braun AG, Melsungen, Germany) into pooled or individual aliquots, which were stored at −80 °C until experimental testing (for a maximum of 3 months). Although the levels of VEGF decreased within 3 months, frozen-stored PRP secretomes demonstrated no negative impact on angiogenesis [24].

In order to validate the preparation of PRP, we compared the platelet number in unfiltered PRP and in the whole blood from three donors using C-Chip hemocytometers (DHC-N01, NanoEnTek Inc., Gyeonggi-do, Korea). We diluted the PRP by 1:400 and the whole blood by 1:200 with PBS and loaded 10 µL of the samples into the hemocytometer chambers. Platelets in the four corners and one small middle square were manually counted under an inverted phase contrast microscope (Axio Vert.A1, Carl Zeiss, Jena, Germany). The platelet concentration was calculated as per the manufacturer’s equation.

### 4.4. Production of Normal Serum (NS)

Donors were identical to the HPS and PRP groups (see Section 4.2). In short, peripheral venous blood was drawn under sterile conditions and collected into separate 30 mL polypropylene syringes (Omnifix^®^, B Braun AG, Melsungen, Germany). For the preparation of normal serum, the syringes were placed upright for 4 h at room temperature to achieve simple sedimentation. Then, the serum supernatant was filtered (Sterifix^®^, B Braun AG, Melsungen, Germany) into a new syringe using the same procedure as HPS (see Section 4.2). The normal serum was stored both separately and pooled at −80 °C until experimental testing.

### 4.5. Cell Culture

Human dermal lymphatic endothelial cells (LECs) from three different donors (*n* = 3) were purchased from PromoCell (C-12217, PromoCell GmbH, Heidelberg, Germany). Cryopreserved cells were thawed and expanded in an expansion medium, which consists of Endothelial Cell Basal Medium MV2 (EBM MV2, PromoCell GmbH, Heidelberg, Germany) supplemented with SupplementMix (C-39226, PromoCell, Heidelberg, Germany), containing a company-stated optimal formula of 5% fetal calf serum (FCS), 5 ng/mL epidermal growth factor (EGF), 10 ng/mL basic fibroblast growth factor (bFGF), 20 ng/mL insulin-like growth factor (IGF), 0.5 ng/mL vascular endothelial growth factor 165 (VEGF-A), 1 μg/mL ascorbic acid, and 0.2 μg/mL hydrocortisone, and were incubated at 37 °C and 5% CO_2_. Ensuing experiments were all carried out between the 3rd–5th cellular passages. The negative control (=basal) media consisted of Endothelial Cell Basal Medium MV2 (EBM MV2, PromoCell GmbH, Heidelberg, Germany) supplemented with 1 μg/mL ascorbic acid, 0.2 μg/mL hydrocortisone, and 1% FCS from the SupplementPack (C-39221, PromoCell, Heidelberg, Germany). For the positive control, we used the expansion media tested and proven by the company to be optimal for LECs. HPS and NS were used as undiluted (100%) and diluted with basal media at 0.1%, 1%, 10%, and 40% final concentrations for the following experiments. PRP was not diluted.

### 4.6. Quantification of the Pro- and Anti-Lymphangiogenic Cytokines

Enzyme-linked immunosorbent assay (ELISA) was utilized to quantify the amount of three key pro-lymphangiogenic (VEGF-C, PDGF-BB, and bFGF) and three anti-lymphangiogenic (TSP-1, PF-4, and endostatin) cytokines in HPS, NS, and PRP from individual donors described in 4.2. (one male donor was not available at the time of secretome production, hence *n* = 9) using the corresponding ELISA kits (DY752B for VEGF-C, DY220 for PDGF-BB, DY233 for bFGF, DY3074 for TSP-1, DY795 for PF-4, DY1098 for endostatin, DuoSet, Bio-Techne Ltd., Minneapolis, MN, USA). The ELISAs were performed according to the manufacturer’s protocols, and optical densities were measured using the Mithras LB 940 Multimode Microplate Reader (Berthold Technologies GmbH & Co. KG, Bad Wildbad, Germany) at a 450 nm wavelength.

### 4.7. Alamar Blue Proliferation Assay

The Alamar Blue assay was used to determine the cell proliferation of LECs. LECs were seeded on 96-well plates at a density of 10,000/cm^2^ in 150 µL of basal medium overnight. On the next day, the medium was replaced by sample media (0.1%, 1%, 10%, 40%, 100% of HPS/NS, and 100% PRP), expansion medium (positive control), and basal medium (negative control), and the cells were incubated for 96 h. Then, the culture media were discarded, and 150 μL of PBS supplemented with 1/10 volume of Alamar Blue solution was added. The plates were incubated at 37 °C, 5% CO_2_ for 4 h. The optimal incubation time has been determined in a preliminary experiment in which the absorbance was measured every 30 min to obtain all values in the measurement scale. Then, 100 µL of the supernatant was transferred to another 96-well plate, and its fluorescence intensity was measured using the Mithras LB 940 Multimode Microplate Reader (Berthold Technologies GmbH & Co. KG, Bad Wildbad, Germany) at an excitation of 560 nm, an emission of 590 nm and a reference wavelength of 629 nm.

### 4.8. Cell Migration Assay

LEC migration assay was performed using culture inserts with a cell-free gap of 500 µm as described by the manufacturer (Ibidi GmbH, Gräfelfing, Germany). In short, 70 µL of LEC suspension solution (8800 cells) reconstituted in the basal medium was seeded into each chamber of the inserts, which have been adhered to the bottom of 24-well plates (an extra 400 μL of the basal medium was added outside of each insert) and incubated at 37 °C and 5% CO_2_ overnight. The next day, the inserts and the spent media were removed, while unattached cells were rinsed off, and the LECs were incubated with 1 mL of the sample media (0.1%, 1%, 10%, 40%, and 100% of HPS/NS, and 100% PRP), expansion medium (positive control) and basal medium (negative control). LEC migration was determined by microscopic imaging at 0 h, 12 h, and 24 h. The culture medium was not changed over the time course of the experiment. The percentage of the cell-free area (=open area) was quantified using the image analysis “Wimscratch tool” (Wimasis, Munich, Germany). For each LEC donor (*n* = 3), the mean of triplicates was calculated.

### 4.9. Tube Formation Assay

The lymphangiogenic potential of the blood-derived secretomes was tested in an in vitro lymphangiogenesis assay by assessing their ability to induce tube formation of LECs. In short, LECs were starved overnight in basal medium, followed by resuspension in sample media (0,1%, 1%, 10%, 40%, and 100% of HPS/NS, and 100% PRP), expansion medium (positive control) and basal medium (negative control). Then, 50 μL of the cell-medium mixture was seeded to μ-Slides (81506, Ibidi GmbH, Martinsried, Germany) precoated with 10 μL of growth factor-reduced Matrigel (356231, Corning Inc., Corning, NY, USA) at a density of 40,000/cm^2^. After 8 h of incubation (37 °C and 5% CO_2_), images from each well were captured by an inverted phase contrast microscope (Axio Vert.A1, Carl Zeiss, Jena, Germany). To identify the living LEC proliferation, cells were stained with 1 μg/mL Calcein AM (C3099, Life Technologies corp., Oregon, USA) in 1× DPBS with Calcium and Magnesium (D1283, Sigma-Aldrich Chemie GmbH, Steinheim, Germany). Images were captured using an inverted fluorescence microscope (Zeiss Axio Observer Z1, Oberkochen, Germany) via the GFP channel. The extent of capillary-like network formation was assessed by the image analysis “WimTube tool” (Wimasis, Munich, Germany), which counted the number and total (cumulative) length of tubes, the number of branching points (point of intersection of two or more tubules), and the cell covered area from four high-power fields (HPFs) taken per well. The mean value of the HPFs was then calculated per well. All conditions were tested in triplicates per LEC donor, and a total of three donors were taken for final evaluation.

### 4.10. Lymphatic Ring Sprouting Assay

Blood-derived secretomes were tested in a lymphatic ring sprouting assay to assess the three-dimensional lymph vessel growth. As previously described [26,42], the thoracic duct from 8–12 weeks old female CD1 mice (Charles River Laboratories, Wilmington, MA, USA) was dissected carefully and cut into 20–25 pieces of less than 0.5 mm in length. The tissue pieces were temporally kept in DMEM (PAN BIOTECH, Aidenbach, Germany) supplemented with 1% antibiotic/antimycotic solution (Capricorn Scientific GmbH, Ebsdorfergrund, Germany) for less than 3 h (37 °C, 5% CO_2_) before the 3D culture. This ex vivo 3D culture eliminates any interferences from inflammatory cells and mimics the in vivo growth with only primary LECs. Briefly, we diluted the collagen type I rat tail (08–115, Sigma-Aldrich, St. Louis, MO, USA) to 1.5 mg/mL by 10× DMEM (D2429, Sigma-Aldrich, St. Louis, MO, USA) and ultra-pure water, and adjusted its pH to neutral. The collagen was dropped on a pre-chilled 96-well plate (50 μL/well) and allowed to polymerize at 37 °C for 20 min. Then, we placed thoracic duct pieces on the collagen surface and covered the tissue with another 50 μL of collagen to form a sandwich-like gel structure. All procedures related to unpolymerized collagen were performed on ice. The plate was then incubated again at 37 °C. After 30 min, 150 μL of the sample media (HPS/NS-10%, -40%, and PRP), expansion medium (positive control), and basal medium (negative control) were added, and the medium was changed every 3 days. On day 11, the outgrowth of sprouts was checked, and images of each complete lymphatic ring were captured by an inverted phase contrast microscope (Axio Vert.A1, Carl Zeiss, Jena, Germany) with different microscope-focus adjustments in order to clearly identify sprouts in different depths. The images were digitally analyzed using ImageJ software (version 1.52v, NeuronJ plugin, National Institutes of Health, Bethesda, MD, USA). From three different mice, each condition was run in triplicates.

### 4.11. Lymphatic Ring Immunostaining

We validated the lymphatic origin of the harvested tissue and vessel sprouts by immunohistochemical anti-LYVE-1-staining as previously described [26,42]. In short, thoracic duct rings embedded in collagen type I were washed and then fixed by Antigenfix (Diapath S.p.A, Martinengo, Italy) at room temperature for 2 h, followed by three washes with PBS. Nonspecific binding sites were blocked using 20% normal goat serum (Abcam, Cambridge, United Kingdom) and 0.2% Triton X-100 (Sigma-Aldrich, St. Louis, MO, USA) in PBS for 1.5 h at room temperature. The samples were then incubated with 3 μg/mL of the rabbit anti-LYVE1 antibodies (Abcam, Cambridge, United Kingdom) diluted by 5% normal goat serum in 0.2% PBST at 4 °C overnight. On the following day, the samples were washed with 0.2% PBST three times and incubated with 1:400 dilution of goat anti-rabbit Alexa488 antibody (Abcam, Cambridge, United Kingdom) at room temperature for 2 h. Nonspecific binding antibodies were washed off with 0.2% PBST. The nuclei were counter-stained with 1:20,000 DAPI solution (D3571, Molecular Probes Inc., Eugene, OR, USA) for 1 h. Fluorescent images were captured in DAPI and GFP channels with an inverted fluorescence microscope (Zeiss Axio Observer Z1, Germany).

### 4.12. Statistical Analysis

Data sets were analyzed by repeated measures of one-way analysis of variance (ANOVA), with subsequent comparisons using Tukey’s post hoc analysis. All values are expressed as means ± standard error of the mean (SEM). A value of *p* < 0.05 was considered statistically significant (* *p* < 0.05, ** *p* < 0.01, *** *p* < 0.001, and **** *p* < 0.0001).

## 5. Conclusions

HPS and PRP are both potent stimulators of LEC activity, each offering different lymphangiogenic potential. Our findings demonstrate the regenerative potential of HPS and PRP in the context of LEC proliferation, migration, tube formation, and lymphatic vessel sprouting. HPS was found to outperform PRP in all mentioned categories, which we believe is due to HPS’s harness of a varied growth factor cocktail secreted from all the peripheral blood cells rather than PRP’s single focus on platelets. The given data may result in positive clinical implications that promote the need for continued research on HPS and PRP in wound healing, scar improvement, and lymphedema.

## 6. Patents

Device-based methods for localized delivery of cell-free carriers with stress-induced cellular factors. (AU2013214187 (B2); 9 February 2017): Schilling Arndt, Hadjipanayi Ektoras, Machens Hans-Günther.

## Data Availability

The data presented in this study are available on request from the corresponding authors.

## Abbreviations

bFGF	Basic fibroblast growth factor
HPS	Hypoxia Preconditioned Serum
IGF	Insulin-like growth factor
LEC	Lymphatic endothelial cell
LYVE-1	Lymphatic vessel endothelial hyaluronan receptor-1
MMP-9	Matrix metalloproteinase-9
PBC	Peripheral blood cells
PBS	Phosphate-buffered saline
PDGF-BB	Platelet-derived growth factor-beta polypeptide
PF-4	Platelet factor-4
PRP	Platelet-rich plasma
TGF-beta	Tumor growth factor-beta
TSP-1	Thrombospondin-1
VEGF	Vascular endothelial growth factor
